# 

**Published:** 1891-07

**Authors:** 


CONTENTS--JULY, 1891.—VOL. VII., NO. 1.

ORIGINAL ARTICLES—

The Ætiology and Rational Treatment of Tetanus; with Cases. Dr. Geo. H. Lee, Galveston.................. 1
How we Treat Wounds to-day. Dr. F. S. White, Terrell 9
Chloralamid in Surgery. Dr. E. Lanphear, Kansas City 12

SOCIETY NOTES—

Austin District Medical Society: Amputation of Cervix Uteri, Discussion on; New Operation for Hernia; Empyema, Discussion on; Typhlitis, Discussion on, etc... 14
Cherokee County Medical Society.................... 24
Texas State Medical Association, note from Secretary.... 25

EDITORIAL—

The New Texas Medical College...................... 27
The Relation of Contract Surgeons of the Several Railroads, to the General Profession.................... 29
The Bug Under the Chip.............................. 30

MEDICAL NEWS AND MISCELLANY—

Numerous items...................................... 31

PUBLISHER'S NOTES..................................... 38

